# The Role of Neck Imaging Reporting and Data System (NI-RADS) in the Management of Head and Neck Cancers

**DOI:** 10.3390/bioengineering12040398

**Published:** 2025-04-08

**Authors:** Daniele Vertulli, Marco Parillo, Carlo Augusto Mallio

**Affiliations:** 1 Radiology Departement, Istituto Dermatologico dell’Immacolata IRCCS, 00167 Rome, Italy; 2 Radiology, Multizonal Unit of Rovereto and Arco, APSS Provincia Autonoma Di Trento, 38123 Trento, Italy; marco.parillo@apss.tn.it; 3 Research Unit of Radiology, Department of Medicine and Surgery, Università Campus Bio-Medico di Roma, 00128 Rome, Italy; c.mallio@policlinicocampus.it

**Keywords:** radiology, oncology, head and neck neoplasms, magnetic resonance imaging, computed X-ray tomography, positron emission tomography, practice guidelines, NI-RADS, narrative review

## Abstract

This review evaluates the current evidence on the use of the Neck Imaging Reporting and Data System (NI-RADS) for the surveillance and early detection of recurrent head and neck cancers. NI-RADS offers a standardized, structured framework specifically tailored for post-treatment imaging, aiding radiologists in differentiating between residual tumors, scar tissue, and post-surgical changes. NI-RADS demonstrated a strong diagnostic performance across multiple studies, with high sensitivity and specificity reported in detecting recurrent tumors at primary and neck sites. Despite these strengths, limitations persist, including a high frequency of indeterminate results and variability in di-agnostic concordance across imaging modalities (computed tomography, magnetic resonance imaging (MRI), positron emission tomography(PET)). The review also highlights the NI-RADS’s reproducibility, showing high inter- and intra-reader agreements across different imaging techniques, although some modality-specific differences were observed. While it demonstrates strong diagnostic performance and good reproducibility across imaging modalities, attention is required to address indeterminate imaging findings and the limitations of modality-specific variations. Future studies should focus on integrating advanced imaging characteristics, such as diffusion-weighted imaging and PET/MRI fusion techniques, to further enhance NI-RADS’s diagnostic accuracy. Continuous efforts to refine NI-RADS protocols and imaging interpretations will ensure more consistent detection of recurrences, ultimately improving clinical outcomes in head and neck cancer management.

## 1. Introduction

Head and neck cancer represents a significant clinical challenge, with high rates of recurrence contributing to poor patient outcomes [1,2]. Detecting recurrent tumors early through non-invasive surveillance imaging is crucial, as early intervention offers better prognostic opportunities. However, imaging-based detection of recurrence is often difficult due to the complex head and neck anatomy, the diversity of treatment modalities, and the variable post-treatment changes that can obscure tumor presence [3]. Treating neck neoplasms necessitates a collaborative approach from a team of medical experts. This team typically includes specialists such as a head and neck surgeon, a radiation oncologist, a medical oncologist, and a radiologist. By working together, these specialists can determine the most effective treatment strategy, which may involve a combination of surgery, chemotherapy, and/or radiotherapy. In this context, radiologists must recognize imaging alterations that result from treatments, as these changes differ across anatomical sites, and they must be able to effectively communicate the imaging findings to the referring physicians. Awareness of these variations, along with knowledge of regions more susceptible to recurrence, is essential to accurately identify tumor recurrence and guide effective treatment planning [4].

The increasing utilization of Reporting and Data Systems (RADS) reflects a trend toward standardized reporting practices within radiology. The goal of these systems is to minimize discrepancies and uncertainties in imaging reports by implementing uniform terminology and structural organization [5,6,7,8]. In line with this trend, the Neck Imaging Reporting and Data System (NI-RADS) was developed by the American College of Radiology [9] to provide a standardized and structured framework for the evaluation of post-treatment imaging in patients with head and neck malignancies [10,11,12]. Unlike other reporting systems such as Breast Imaging Reporting and Data System (BI-RADS) or Liver Imaging Reporting and Data System (LI-RADS), which address diagnostic or screening purposes, NI-RADS is specifically tailored for post-treatment surveillance, ensuring consistent communication among physicians.

The present review assesses the available evidence regarding NI-RADS in the context of surveillance and early detection of recurrent head and neck malignancies, with particular attention to its diagnostic efficacy and consistency. The observations and conclusions drawn herein will serve to guide subsequent research endeavors targeting the less-understood elements of the NI-RADS scoring methodology.

## 2. Methods

**Figure 1:**
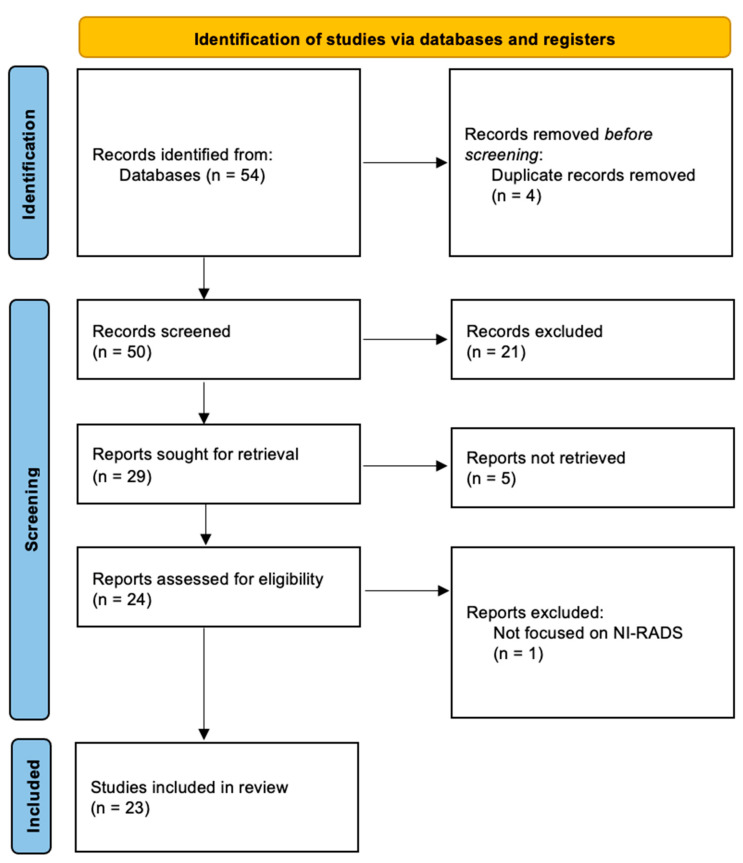
The PRISMA 2020 flow chart for the article-selection process.

We conducted a comprehensive literature review by searching the Scopus, MEDLINE, and PubMed Central databases. The search included all relevant articles indexed in these platforms up to 1 December 2024. To identify pertinent studies, we employed the search terms: “NI-RADS” OR “Neck Imaging Reporting and Data System”, using the PRISMA guidelines flowchart (Figure 1). Only English-language publications were considered to ensure a clear interpretation of results. Our focus was on original research articles, excluding reviews, editorials, and case reports, to prioritize the latest and most robust findings. After an initial examination of titles and abstracts by a junior radiologist (D.V.), we selected 23 relevant articles from an initial set of 50 A thorough review of these full texts, along with cross-referencing, was performed to ensure a comprehensive evaluation of the current literature landscape. We did not include any studies specifically related to Artificial Intelligence (AI) and NI-RADS.

## 3. NI-RADS Score

NI-RADS is applicable for monitoring several head and neck cancers using clinical imaging. These include squamous cell carcinomas affecting the skin of the head and neck, as well as those arising in the oral cavity, nasal cavity, nasopharynx, oropharynx, hypopharynx, and larynx. NI-RADS is also used for surveillance of non-squamous cell head and neck cancers, such as salivary gland tumors, sinonasal tumors, orbital tumors, and thyroid cancer.

NI-RADS uses a four-tier classification system (NI-RADS 0 to 4, see Table 1), with each category corresponding to a specific level of suspicion for recurrence and associated management recommendations. For instance, NI-RADS 1 indicates no evidence of recurrence and suggests routine surveillance, whereas NI-RADS 3 signals high suspicion and typically recommends biopsy. This approach is designed to streamline decision-making and reduce ambiguity in follow-up strategies.

The system is primarily intended for contrast-enhanced computed tomography (CECT) or magnetic resonance imaging (MRI), with or without positron emission tomography (PET) imaging with 2-[(18)F]fluoro-2-deoxy-D-glucose (FDG), to evaluate both the primary tumor site and regional lymph nodes (neck site). It is important to note that CT and MRI utilize separate lexicons and risk assessment frameworks, each tailored to the specific imaging modality [13]. These frameworks incorporate specific imaging characteristics, such as mucosal enhancement, nodal morphology, and FDG uptake patterns, to refine risk stratification and facilitate standardized reporting across institutions using NI-RADS scoring.

## 4. The NI-RADS’ Diagnostic and Prognostic Value

The diagnostic performance of NI-RADS has been assessed in a variety of head and neck cancers since its introduction. In clinical settings, a robust radiological classification system is needed to reliably distinguish between the effects of treatment and the recurrence of disease (see Table 2).

Dinkelborg et al. [13] reported recurrence rates for the primary site: 1.0% (NI-RADS 1), 7.1% (2a), 5.6% (2b), 66.7% (3), and 100.0% (4). For the neck, rates were 0.5% (1), 7.0% (2), 80.0% (3), and 100.0% (4). NI-RADS demonstrated high area under the curve (AUC) values: 0.934 (primary site) and 0.959 (neck).

A separate study [14] analyzing 318 scans and 618 targets revealed that NI-RADS categories effectively stratified disease recurrence risk: 3.79% for NI-RADS 1, 17.2% for NI-RADS 2, and 59.4% for NI-RADS 3. They demonstrated strong associations between higher NI-RADS scores and increased likelihood of recurrence (*p* < 0.001).

As reported by Kumar et al. [29], CECT alone can effectively assign NI-RADS scores to predict recurrent tumors in neck malignancy patients. Disease persistence at the primary tumor site showed rates of 4% for NI-RADS 1, 24% for NI-RADS 2, and 80% for NI-RADS 3. Moreover, the NI-RADS categories showed nodal recurrence rates of 5.3% (1), 25% (2), and 66.7% (3).

In a study [22] of 220 patients with stage III to IVB oral squamous cell carcinoma (OSCC), the NI-RADS classification of PET/CT scans three months post-adjuvant therapy demonstrated a strong association with locoregional progression, distant metastases, and overall survival. Patients with suspicious scans (NI-RADS 3 or higher) had a significantly higher risk of local failure (HR 14.0, 95% CI 7.3–26.6), distant failure (HR 18.4, 95% CI 9.6–35.3), and poorer overall survival (HR 9.5, 95% CI 5.0–17.9) compared to those with non-suspicious scans. The system’s sensitivity (58%) and specificity (92%) underline its value in detecting actionable recurrences, although its predictive power remains imperfect.

Paul et al. [33] validated the diagnostic performance of NI-RADS in post-treatment FDG-PET/CECT scans, where it demonstrated high diagnostic accuracy for detecting locoregional recurrence. Specifically, diagnostic sensitivity, specificity, and positive predictive value (PPV) were 73.5%, 81.4%, and 46.3% at the primary site, while at the neck, the corresponding metrics were 72.7%, 87.5%, and 43.2%. Combining primary site and neck, the corresponding metrics of diagnostic accuracy were 84.4%, 69.7%, 46.3%, 93.5%, and 73.2%, respectively.

Wangaryattawanich et al. [21] reported a PPV of 56% for NI-RADS 3 and 100% for NI-RADS 4 on PET/CT imaging, highlighting its capacity to differentiate between suspicious and definitive recurrence accurately. FDG-PET/CT also demonstrates a high negative predictive value (NPV) in NI-RADS categories; indeed, patients with incomplete response (NI-RADS 2) showed an NPV of 85% for 2-year disease-free survival, underscoring the need for more rigorous follow-up compared to NI-RADS 1 patients [16]. Johansson et al. [31] observed that 71% of NI-RADS 3 lesions represented recurrences, reinforcing their significance in identifying high-risk cases. Paul et al. [33] demonstrated superior 2-year locoregional control rates for patients categorized as NI-RADS 1 (94.2%) compared to those in higher categories (category 2: 69.4%; category 3: 20.4%).

Mahajan et al. [32] demonstrated that the concordance and accuracy of NI-RADS vary across categories, with higher accuracy observed in NI-RADS 1 and NI-RADS 4 (100% and 82%, respectively, for primary sites); moreover, they showed a variability in the use of different imaging modalities. PET/CT showed higher concordance (91%) in primary NI-RADS 2 compared to CECT (55%), whereas CECT had superior concordance (57%) in primary NI-RADS 3 over PET/CT (41%). The NI-RADS lexicon demonstrated higher accuracy at nodal sites compared to primary sites.

According to Hsu et al. [17], the increasing NI-RADS category correlates with an elevated likelihood of treatment failure. This study analyzed 199 patients followed for a median of 15.5 months post-treatment. Treatment failure rates increased with higher NI-RADS categories (4.3%, 9.1%, and 42.1%). A Cox model showed a significant correlation between NI-RADS categories and treatment failure at primary and neck sites (HR, 2.60 and 5.22; *p* < 0.001). However, in the surgical subgroup, the association between NI-RADS category and treatment failure at the primary site was not statistically significant (HR, 0.88; *p* = 0.82).

Several studies indicate the benefit of integrating additional imaging characteristics in MRI to improve NI-RADS diagnostic accuracy. For instance, a retrospective analysis [35] revealed recurrence rates of 90% for NI-RADS 3 and 45% for NI-RADS 2, indicating the robustness of these categories in stratifying recurrence risks. They demonstrated that integrating diffusion-weighted imaging (DWI) and apparent diffusion coefficient (ADC) improved sensitivity (88.9%) and overall diagnostic accuracy (85.1%), outperforming the standard NI-RADS lexicon. As reported by Ashour et al. [25], integrating DWI and T2 signal criteria enhances diagnostic performance with sensitivity of 92.3% (vs. 84.6% with standard NI-RADS), specificity of 90.7% (vs. 81.4%), PPV of 85.7% (vs. 73.3%), NPV of 95.1% (vs. 89.8%), and accuracy of 91.3% (vs. 82.6%). Similarly, a study [26] using PET/MRI highlighted an AUC of up to 0.987 for predicting treatment outcomes.

NI-RADS is also comparable to other interpretative criteria like Porceddu, Hopkins, and Deauville but exhibits a higher frequency of indeterminate results, as reported in Zhong et al. [18].

Lee et al. [28] demonstrated its effectiveness even when used by general neuroradiologists, with an AUC of 0.765 for primary site predictions and 0.820 for lymph nodes.

In a retrospective analysis [34] of patients undergoing salvage total laryngectomy with elective neck dissection, 81 individuals were evaluated using NI-RADS prior to surgery. Notably, 16% exhibited occult nodal disease (OND) on pathology despite most being scored NI-RADS 1. Only one patient with OND scored NI-RADS 2. Moreover, preoperative imaging re-analysis revealed that only 31% of occult nodes displayed suspicious features on CT, such as rounded morphology, and most PET scans demonstrated SUVmax below blood pool levels [34]. These challenges suggest that NI-RADS reliability can be undermined by subtle disease presentation. Conversely, in OSCC, the specificity of 92% in identifying disease recurrence validates its use as a reliable tool for post-treatment surveillance [22]. However, reliance on imaging alone for definitive conclusions can lead to missed diagnoses, as illustrated in the OND cases.

As reported by Hiyama et al. [36], the NI-RADS score provides a structured way of categorizing these findings, considering areas prone to recurrence, such as the resection margins, the reconstructed flap margins, and deep anatomic sites. By assigning appropriate NI-RADS scores, radiologists can better differentiate between scar tissue, post-surgical changes, and residual tumors, thus ensuring timely and accurate clinical decisions.

In summary, multiple studies validate NI-RADS diagnostic performance, demonstrating strong correlations between higher NI-RADS scores and increased recurrence risk in head and neck cancers. Recurrence rates for primary site and neck vary significantly across NI-RADS categories, with NI-RADS 3 and 4 exhibiting the highest likelihoods of recurrence (e.g., 66.7–100%). A meta-analysis [30] of seven studies (694 patients, 1233 lesions) estimated recurrence rates of 74.4% (NI-RADS 3), 29.0% (NI-RADS 2), and 4.2% (NI-RADS 1) for primary lesions, and 73.3%, 14.3%, and 3.5% for cervical lymph nodes, respectively. NI-RADS 3 demonstrated superior diagnostic accuracy, with an AUC of 0.887–0.983. CECT alone effectively assigns NI-RADS scores, predicting tumor recurrence with high specificity. PET/MRI improves sensitivity, particularly when incorporating DWI and ADC. Studies report increased sensitivity (92.3%) and specificity (90.7%) with integrated imaging. Despite NI-RADS performance, indeterminate results and occult nodal disease remain challenges. Imaging alone may overlook subtle recurrences, necessitating clinical correlation.

## 5. The NI-RADS’s Reliability

To ensure its consistent application, NI-RADS, similar to other RADS scores, must be validated through studies assessing inter-rater reliability. Inconsistent interpretation, reflected in low inter-rater agreement, could diminish the system’s value in clinical practice [5,6].

In the study by Abdelaziz et al. [19], high inter-reader agreement was observed for the final NI-RADS category of primary lesions and lymph nodes (K = 0.808 and 0.806, respectively). However, reproducibility was superior with CT compared to MRI (K = 0.843 vs. 0.77, *p* = 0.001), suggesting modality-specific variability in the application of the lexicon. Almost perfect agreement was seen in excluding tissue enhancement (K = 0.826, 95% CI = 0.658–0.993, *p* = 0.001) and substantial agreement for discrete nodular and diffuse mucosal enhancement (K = 0.826, 95% CI = 0.658–0.993, *p* = 0.001). Fair agreement was noted for focal mucosal nonmass and deep ill-defined enhancement. Intra-reader agreement was almost perfect across most features (K = 0.802–1), except for enlarging discrete nodule/mass and focal mucosal nonmass-like enhancement, which showed substantial intra-observer agreement (K = 0.768–0.786).

As reported by Elsholtz et al. [23], inter-reader agreement for NI-RADS categories on contrast-enhanced MRI datasets showed substantial reliability for neck assessments (κFleiss = 0.67), while inter-reader agreement for the primary site was moderate (κFleiss = 0.53). Notably, the agreement was higher for DWI sequences, where κFleiss reached 0.83. Dinkelborg et al. [13] reported inter-reader agreement of 0.67 for primary site and 0.81 for neck.

Elsholtz et al. [20] evaluated NI-RADS’s reproducibility and found strong inter-reader agreement at the primary site (Kendall’s W = 0.74) and cervical nodes (W = 0.80). Intra-reader agreement was similarly robust, with weighted kappa values ranging from 0.85 to 0.96. These findings demonstrate that NI-RADS provides consistent and reproducible results across radiologists, although challenges remain in cases complicated by post-treatment changes without recurrence.

Hsu et al. [24] assessed inter-rater reliability across eight radiologists from different institutions using post-treatment FDG-PET/CECT scans. Despite variability in experience (some raters having more than five years of experience and others less), the NI-RADS achieved moderate inter-observer agreement at the primary site (Light k = 0.55) and higher agreement at the neck site (Light k = 0.60). This study emphasizes that while moderate inter-reader variability remains, the NI-RADS is a reliable tool across different clinical settings and among radiologists with varying expertise levels.

Additionally, supervision by subspecialized radiologists improved specificity and PPV, particularly for higher-risk categories, as shown by Elsholtz et al. [27]. They analyzed 150 CT and MRI datasets initially reviewed by two trained residents and subsequently supervised by two subspecialized radiologists. Supervising radiologists modified 26% of the initial reports. Receiver operating characteristic analysis showed higher AUC values for the supervision session compared to the initial reading for both the primary site (0.89 vs. 0.86) and the neck (0.94 vs. 0.91), though differences were not statistically significant. Dichotomized NI-RADS categories revealed statistically significant improvements in specificity and PPV for the primary site and both sites combined during the supervision session.

In summary, high inter-reader agreement has been observed for primary lesion and lymph node NI-RADS categories, with superior reproducibility using CT compared to MRI, indicating modality-dependent variability. Strong agreement has been reported for tissue enhancement exclusion, and substantial agreement for nodular and diffuse mucosal enhancement. Intra-reader agreement is nearly perfect across most features. Inter-reader reliability for NI-RADS categories on contrast-enhanced MRI has shown substantial agreement for neck assessments but moderate agreement for the primary site. Higher agreement has been noted for DWI sequences. Studies have reported inter-reader agreement ranging from moderate to strong for primary and neck sites, confirming NI-RADS’s reproducibility. Moderate agreement has also been observed among radiologists analyzing post-treatment FDG-PET/CECT scans, demonstrating the system’s reliability across different clinical settings. Supervision by subspecialized radiologists improves specificity and PPV, particularly in high-risk categories. In supervised assessments, a significant proportion of initial reports are modified, with increased AUC values for primary and neck sites. These findings highlight the benefits of expert review in optimizing NI-RADS performance.

## 6. The NI-RADS’ Report Quality and Acceptance by Physicians

Patients with head and neck tumors often require frequent management adjustments, which are typically based on the interpretation of imaging studies. These interpretations are often made within the context of multidisciplinary teams and can lead to revisions in the initial diagnosis, inform surgical strategies, and guide therapeutic planning. Therefore, it is crucial that NI-RADS achieve broad approval and recognition among all physicians participating in the multidisciplinary care of these patients.

Bunch et al. [30] reported that 100% of referring physicians found NI-RADS reports clear, understandable, and helpful for clinical management. Similarly, 88% of radiologists agreed that NI-RADS improved consistency in reporting. Over three months, NI-RADS utilization among radiologists increased from 46% to 72%, reflecting growing familiarity and trust in the system. This highlights the system’s effectiveness in improving communication and decision-making in multidisciplinary teams.

NI-RADS facilitates effective communication among healthcare providers by stratifying recurrence risk into actionable categories. As reported by Paul et al. [33], physicians were able to stratify locoregional recurrence based on NI-RADS categories, which facilitated decision-making about treatment modifications and follow-ups.

Dinkelborg et al. [13] demonstrate a commitment among clinicians to use the NI-RADS in surveillance protocols for postoperative OSCC.

NI-RADS integration into surveillance protocols for OSCC demonstrated improved identification of recurrence compared to non-standardized imaging interpretations in earlier studies [22]. Nonetheless, the variability in sensitivity and reliance on clinical expertise in interpreting subtle findings, such as in OND, highlights a need for improved training and refinement of reporting criteria [34].

In summary, preliminary findings suggest that the NI-RADS structured reporting system contributes to improved communication within multidisciplinary teams responsible for the care of patients with head and neck tumors. The standardized format promotes the clear and concise transmission of all necessary information among the specialists involved.

## 7. Future Perspectives

Further research is needed to evaluate CECT performance in NI-RADS 3 and 4 categories and to determine the utility of PET/CECT for close interval follow-ups in stage III/IV NI-RADS 2 cases [32]. Patel et al. [26] underscore the potential role of hybrid PET/MRI in high-risk patient groups.

As documented by Elsholtz et al. [23], DWI showed superior inter-reader agreement for the primary site, suggesting its potential as an additional reliable criterion to complement NI-RADS assessments.

Wangaryattawanich et al. [21] highlighted the relatively low PPV of NI-RADS 3 on PET/CT (56%), suggesting the need for confirmatory tissue sampling to prevent overtreatment. Tailored recommendations based on evolving imaging techniques and clinical contexts could improve outcomes and reduce unnecessary interventions. Integrating advanced imaging markers, such as DWI, T2-weighted imaging, and ADC parameters, has shown promise [35].

Future research should focus on integrating NI-RADS with emerging imaging modalities and AI tools to further enhance predictive accuracy and reduce variability. AI-driven tools could assist radiologists in assigning NI-RADS categories, particularly in scenarios with high inter-observer variability. Looking ahead, large language models could potentially be used to automatically classify NI-RADS based on detailed radiological report descriptions, further improving standardization and diagnostic precision [15]. Research into broader population cohorts across diverse healthcare systems and multicenter studies, such as in the study of Hsu et al. [24], would help standardize NI-RADS application globally, ensuring universal applicability and clinical utility.

Additionally, patient-centered approaches in radiology reporting, as mentioned by Mohan et al. [37], may complement NI-RADS by improving patient engagement and understanding of post-treatment imaging. More patients wanted communication from the radiologist (26% to 44%) or both specialists (19% to 33%). A total of 70–93% of patients gained a better understanding of imaging findings and follow-up recommendations. A total of 93% expressed interest in future consultations with a radiologist, and 96% found the session helpful.

Furthermore, hyperspectral imaging (HSI) represents a promising advancement in oncologic imaging, offering a non-invasive method to enhance tumor detection and characterization through detailed spectral analysis. Recent research has highlighted its potential to improve the differentiation between malignant and benign tissues by leveraging unique spectral signatures that are imperceptible in conventional imaging [38,39]. The integration of HSI with NI-RADS could refine risk stratification by providing additional quantitative data to support radiological assessments. Combining HSI with AI-based diagnostic algorithms may further enhance its clinical applicability, enabling automated lesion classification and more precise treatment planning. As technology matures, its role within structured reporting frameworks like NI-RADS warrants further exploration to optimize oncologic imaging workflows.

## 8. A Summary of the NI-RADS’ Pros and Cons

The NI-RADS framework offers substantial diagnostic and prognostic utility across multiple imaging modalities, such as FDG-PET/CECT and CECT scans. The robust stratification capabilities of NI-RADS, with metrics demonstrating notable sensitivity, specificity, and predictive values, underscore its clinical relevance. NI-RADS provides a structured and standardized approach to categorizing findings, facilitating clear communication among multidisciplinary teams. It enhances diagnostic accuracy, particularly in differentiating scar tissue from residual tumors. Additionally, it is supported by high inter- and intra-reader reproducibility, ensuring consistency across radiologists and imaging modalities. Studies show improved decision-making among physicians, with NI-RADS aiding treatment planning and follow-up protocols. In this scenario, broad awareness and adoption of NI-RADS among all physicians involved in head and neck tumor follow-up are essential. Including a score in the radiology report without consensus from other physicians could create more confusion than benefit. However, even if the final NI-RADS category is not reported, the system serves as a valuable framework for interpreting surveillance images of head and neck patients and, as such, warrants widespread knowledge.

Despite its strengths, NI-RADS also has notable limitations. The frequency of indeterminate results poses a diagnostic challenge. Modality-specific variability in inter-reader agreement, especially between CT and MRI, further complicates evaluations. Additionally, cases with post-treatment changes without evident recurrence remain difficult to interpret. Addressing these limitations will require incorporating more advanced imaging features like DWI and AI tools for improved predictive accuracy and standardization across diverse clinical settings.

Table 2 presents an overview of the primary research articles examining the application of NI-RADS in clinical practice.

## Figures and Tables

**Table 1:** Summary of the NI-RADS, outlining imaging findings and corresponding management recommendations for head and neck cancer surveillance. FDG, 2-[(18)F]fluoro-2-deoxy-D-glucose; PET, positron emission tomography; CT, computed tomography; MRI, magnetic resonance imaging.

Score	Imaging Findings		Management
	Primary Site	Neck Site	
0	Incomplete study (prior imaging unavailable)		Obtain prior imaging and reassess.
1	Expected anatomical changes following treatment	Hypoenhancing residual nodal tissue without FDG uptake (if PET available)	Continue routine surveillance.
2a for Primary Site	Superficial mucosal abnormality with mild enhancement	-	Direct visual inspection.
2b for Primary Site and 2 for Neck Site	Deep non-nodular, ill-defined soft tissue abnormality	Potential residual disease, including heterogeneous enhancement or mild/moderate FDG uptake in residual nodal tissue (if PET is available), new or enlarging lymph nodes without definitively abnormal morphology, and any PET/CT/MRI discordance	Close follow-up (3 months) with MRI/CT or PET to evaluate suspicious nodes or deep submucosal abnormalities.
3	Discrete primary site nodule/mass with intense focal FDG uptake	Concerning nodal findings, including intense FDG uptake or enlargement/increased enhancement in residual tissue, and necrosis, irregular borders, or focal intense FDG uptake in new/enlarging nodes.	Biopsy of the concerning area.
4	Proven pathological or clear radiologic/clinical disease progression.		Clinical management

**Table 2:** Main articles that have evaluated the role of the NI-RADS in clinical practice.

Investigators	Study Design	Clinical Setting	Number of MRIs/CTs	Main Findings
Krieger et al. (2017) [14]	Retrospective, quality-improvement study	Post-treatment	318 PET-CTs	Strong performance of NI-RADS for predicting disease recurrence, significant discrimination between categories 1–3 (*p* < 0.001)
Mohan et al. (2018) [15]	Prospective	Post-surgery and post RT/CT	27 FDG PET-CTs	Direct patient reporting improved understanding of imaging findings and radiologist′s role (70–93%). Patients preferred radiologist consultation (44%) or combined with the physician (33%).
Wangaryattawanich et al. (2018) [16]	Retrospective	Post RT/CT	110 FDG-PET/CTs	A total of 85% NPV in NI-RADS 2; treatment failure mainly in cervical lymph nodes (15% within 2 years).
Hsu et al. (2019) [17]	Retrospective	Post-surgery and post RT/CT	199 PET/CECTs	NI-RADS categories strongly correlated with treatment failure risk; higher categories show higher failure rates.
Zhong et al. (2020) [18]	Retrospective	Post RT/CT	562 FDG PET/CTs	Compared NI-RADS, Porceddu, Hopkins, and Deauville for predicting loco-regional control and PFS. Porceddu and Deauville minimized indeterminate outcomes.
Abdelaziz et al. (2020) [19]	Retrospective	Post-surgery or Post RT/CT	97 CECT/MRIs (58 patients)	High inter-reader agreement for primary lesions and lymph nodes (K = 0.808, K = 0.806). Better agreement for CT (K = 0.843) than MRI (K = 0.77). Substantial agreement for tissue and mucosal enhancement. Variable confidence in individual features, but overall NI-RADS category is unaffected.
Elsholtz et al. (2020) [20]	Retrospective	Post-treatment	101 CTs	Good inter-reader reproducibility; higher agreement in patients with proven recurrence (W = 0.96, kF = 0.65).Effective intra-reader agreement across primary site and neck (tB = 0.67–0.82, kw = 0.85–0.96).
Wangaryattawanich et al. (2020) [21]	Retrospective	Post-surgery	128 PET/CTs	PPV of NI-RADS 3: 56%; NI-RADS 4: 100%; confirmation needed for NI-RADS 3.
Qian et al. (2020) [22]	Retrospective	Post surgery and post RT/CT	220 PET/CTs	Suspicious scans (30%) predict locoregional failure (HR 14.0), distant failure (HR 18.4), and poorer survival (HR 9.5). Overall PPV: 85%, Sensitivity: 58%, Specificity: 92%. Salvage success rate: 11%.
Elsholtz et al. (2021) [23]	Retrospective	Post-treatment	104 MRIs	NI-RADS inter-reader agreement was moderate (κFleiss = 0.53) for primary site, substantial for neck (κFleiss = 0.67), and high for DWI (κFleiss = 0.83). DWI may improve agreement reliability.
Dinkelborg et al. (2021) [13]	Retrospective	Post-surgery	503 CECT/MRIs	NI-RADS effectively detects OSCC recurrence at the primary site and neck with high AUC values (0.934/0.959)Recurrence rates: 100% in NI-RADS 4, 66.7% in NI-RADS 3. Inter-reader agreement: 0.67–0.81.
Hsu et al. (2021) [24]	Retrospective	Post-treatment	80 PET/CTs	Moderate inter-reader agreement with Light k = 0.55 (primary site) and 0.60 (neck site) using NI-RADS categories.
Ashour et al. (2021) [25]	Retrospective	Post-surgery	69 MRIs	Adding T2 signal and DWI enhances NI-RADS accuracy and specificity.
Patel et al. (2022) [26]	Retrospective	Post RT/CT	46 PET/MRIs	PET/MRI showed substantial inter-reader agreement (κ = 0.634). High diagnostic accuracy for treatment failure (AUC 0.864–0.987).Potential role in surveillance imaging.
Elsholtz et al. (2022) [27]	Prospective	Post-surgery/RT/CT	150 CT/MRIs	A total of 26% of reports were modified after supervision by subspecialized radiologists.Higher ROC AUC with supervision: 0.89 vs. 0.86 (primary site), 0.94 vs. 0.91 (neck).Statistically significant improvement in specificity and PPV after supervision.
Lee et al. (2022) [28]	Retrospective	Post-surgery or post-RT/CT	608 CT/MRIs	NI-RADS categories predict recurrence: primary site (AUC 0.765), lymph nodes (AUC 0.820). Recurrence rates: NI-RADS 1 (5%), 2 (29%), 3 (65%).
Kumar et al. (2022) [29]	Prospective	Post-RT/CT	30 CECTs	NI-RADS 1: 4% persistence; NI-RADS 2: 24%; NI-RADS 3: 80% persistence rates. Nodal recurrence rates: NI-RADS 1: 5.3%, NI-RADS 2: 25%, NI-RADS 3: 66.7%.
Bunch et al. (2022) [30]	Quality improvement	Post-surgery	22 CTs	NI-RADS reports were clear, understandable, and guided clinical management. Radiologists’ reporting consistency improved significantly.
Johansson et al. (2022) [31]	Retrospective	Post-surgery/RT	580 CTs	PPV of NI-RADS 3: 71%
Jajodia et al. (2022) [25]	Retrospective	Post-surgery	61 MRIs	Evaluated NI-RADS categories 2 and 3 with T2WI, DWI, and ADC imaging, showing improved diagnostic accuracy.
Mahajan et al. (2023) [32]	Retrospective	Post RT/CT	462 PET/CTs	NI-RADS accuracy: Nodes (NI-RADS 1–4): 92%, 97%, 90%, 67%. Primary: PET/CT better at NI-RADS2 (91% vs. 55% CECT), CECT better at NI-RADS3 (57% vs. 41% PET/CT).
Paul et al. (2023) [33]	Retrospective	Post RT/CT	190 PET/CECTs	NI-RADS showed high diagnostic accuracy; 2-year locoregional control 94.2% for NI-RADS 1.
Chan et al. (2025) [34]	Retrospective	Post-surgery	81 PET/CTs	A total of 16% had OND; occult nodes were subtle on imaging; most had SUVmax below blood pool on PET; NI-RADS 1 or 2 in all cases.

## Abbreviations

The following abbreviations are used in this manuscript:

AI	artificial intelligence
AUC	Area under the curve
BI-RADS	Breast Imaging Reporting and Data System
CAD-RADS	Coronary Artery Disease Reporting and Data System
CECT	Contrast-Enhanced Computed Tomography
CT	Computed Tomography
DWI	Diffusion-Weighted Imaging
FDG-PET	Fluorodeoxyglucose Positron Emission Tomography
HR	hazard ratio
HIS	hyperspectral imaging
K	kappa test parameter
LI-RADS	Liver Imaging Reporting and Data System
MRI	Magnetic Resonance Imaging
NI-RADS	Neck Imaging Reporting and Data System
NPV	negative predictive value
OND	occult nodal disease
OSCC	oral squamous cell carcinoma
PPV	positive predictive value
RADS	Reporting and Data System
RT/CT	radio-chemotherapy
